# A Unique Mode of Coenzyme A Binding to the Nucleotide Binding Pocket of Human Metastasis Suppressor NME1

**DOI:** 10.3390/ijms24119359

**Published:** 2023-05-27

**Authors:** Maria-Armineh Tossounian, Stefan Denchev Hristov, Jonathan Alexis Semelak, Bess Yi Kun Yu, Maria Baczynska, Yuhan Zhao, Dario Ariel Estrin, Madia Trujillo, Valeriy Filonenko, Jerome Gouge, Ivan Gout

**Affiliations:** 1 Department of Structural and Molecular Biology, University College London, London WC1E 6BT, UK; s.hristov22@imperial.ac.uk (S.D.H.); bess.yu.15@ucl.ac.uk (B.Y.K.Y.); maria.baczynska.22@ucl.ac.uk (M.B.); yuhan.zhao.19@ucl.ac.uk (Y.Z.); i.gout@ucl.ac.uk (I.G.); 2 Departmento de Química Inorgánica Analítica y Química Física, Instituto de Química Física de los Materiales, Medioambiente y Energía (INQUIMAE) and Consejo Nacional de Investigaciones Científicas y Técnicas (CONICET), Ciudad Universitaria, Pab. 2 C1428EHA, Buenos Aires 1865, Argentina; jsemelak@qi.fcen.uba.ar (J.A.S.); dario@qi.fcen.uba.ar (D.A.E.); 3 Departamento de Bioquímica, Facultad de Medicina, Universidad de la República, Montevideo 11800, Uruguay; madiat@fmed.edu.uy; 4 Centro de Investigaciones Biomédicas (CEINBIO), Universidad de la República, Montevideo 11800, Uruguay; 5 Department of Cell Signaling, Institute of Molecular Biology and Genetics, National Academy of Sciences of Ukraine, 03680 Kyiv, Ukraine; filonenko@imbg.org.ua; 6 Institute of Structural and Molecular Biology, Birkbeck College, University of London, London WC1E 7HX, UK

**Keywords:** metastasis suppressor, NME1, coenzyme A, CoAlation, NDPK-A structure, NM23-H1, nucleotide binding, molecular dynamics, X-ray crystallography, protein-metabolite regulation

## Abstract

Coenzyme A (CoA) is a key cellular metabolite which participates in diverse metabolic pathways, regulation of gene expression and the antioxidant defense mechanism. Human NME1 (hNME1), which is a moonlighting protein, was identified as a major CoA-binding protein. Biochemical studies showed that hNME1 is regulated by CoA through both covalent and non-covalent binding, which leads to a decrease in the hNME1 nucleoside diphosphate kinase (NDPK) activity. In this study, we expanded the knowledge on previous findings by focusing on the non-covalent mode of CoA binding to the hNME1. With X-ray crystallography, we solved the CoA bound structure of hNME1 (hNME1-CoA) and determined the stabilization interactions CoA forms within the nucleotide-binding site of hNME1. A hydrophobic patch stabilizing the CoA adenine ring, while salt bridges and hydrogen bonds stabilizing the phosphate groups of CoA were observed. With molecular dynamics studies, we extended our structural analysis by characterizing the hNME1-CoA structure and elucidating possible orientations of the pantetheine tail, which is absent in the X-ray structure due to its flexibility. Crystallographic studies suggested the involvement of arginine 58 and threonine 94 in mediating specific interactions with CoA. Site-directed mutagenesis and CoA-based affinity purifications showed that arginine 58 mutation to glutamate (R58E) and threonine 94 mutation to aspartate (T94D) prevent hNME1 from binding to CoA. Overall, our results reveal a unique mode by which hNME1 binds CoA, which differs significantly from that of ADP binding: the α- and β-phosphates of CoA are oriented away from the nucleotide-binding site, while 3′-phosphate faces catalytic histidine 118 (H118). The interactions formed by the CoA adenine ring and phosphate groups contribute to the specific mode of CoA binding to hNME1.

## 1. Introduction

**Figure 1:**
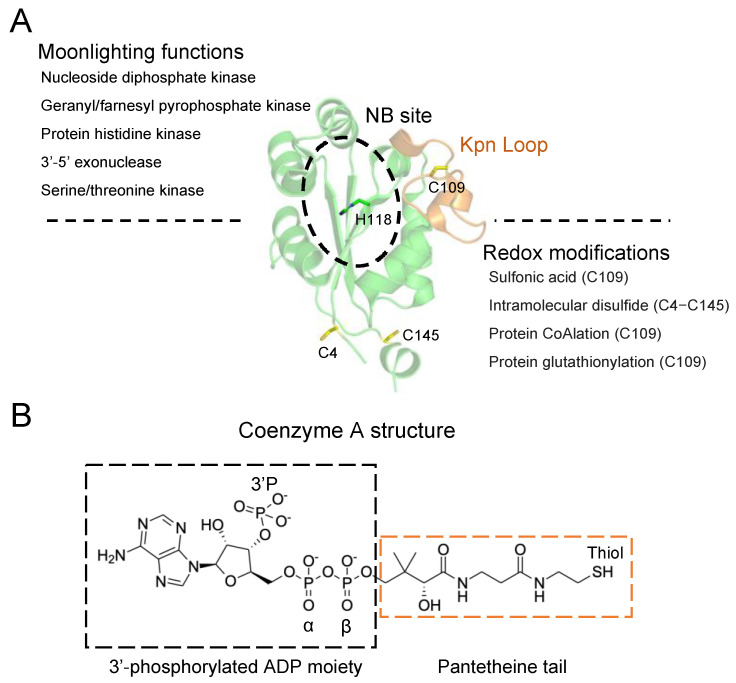
Human NME1 moonlighting functions and redox regulation. (**A**) The different reported functions of human NME1 are shown. The structure of NME1 (PDB: 2HVD) is presented in a green Cartoon, the cysteine residues (C4, C109 and C145) in yellow Sticks, and the catalytic histidine (H118) in green Sticks. The Kpn loop is indicated in orange, and the nucleotide-binding (NB) site is shown in black dashed circle. The different types of redox modifications observed on NME1 are shown and include sulfonylation, CoAlation, glutathionylation and intramolecular disulfide bond formation. (**B**) The CoA structure is composed of a 3′-phosphorylated ADP moiety (black dashed box) and a long pantetheine tail with a reactive thiol group at its terminal (orange dashed box). CoA structure was generated using ChemDraw.

The human NME1 (non-metastatic expressed, isoform H1) is the first identified metastasis suppressor protein that plays a role in the inhibition of one or more steps within the metastatic cascade [1]. NME1 is also known as nucleoside diphosphate kinase A (NDPK-A and NM23-H1). NME1 is a moonlighting protein with multiple catalytic functions: (1) the NDPK activity, which involves the transfer of a γ-phosphoryl group from a nucleoside triphosphate (NTP) to a nucleoside diphosphate (NDP) through the transient phosphorylation of histidine 118 (H118). The reaction occurs through a ping-pong mechanism and uses ATP as its major phosphate donor [2,3]; (2) the protein histidine kinase activity, which involves the transfer of an activated phosphate group from the NME1 autophosphorylated H118 onto a histidine residue on the target protein [4]; (3) the geranyl/farnesyl pyrophosphate kinase activity; (4) 3′-5′ exonuclease activity; and (5) the serine-threonine kinase activity [5,6,7]. Due to its moonlighting activities, NME1 has been shown to participate in diverse cellular functions including the control of intracellular nucleotide homeostasis, cell proliferation, differentiation and development, regulation of gene expression, signal transduction and G-protein-coupled receptor endocytosis [8,9,10,11] (Figure 1A).

NME proteins are present in all living organisms. While most bacteria have a single NME (NDPK) isoform, eukaryotes have been reported to contain multiple isoforms, which are located at different cellular compartments (e.g., cytosol, mitochondria, and nucleus) [12,13,14,15]. Ten human NME homologs have been identified so far, and they are divided into two groups based on their sequence homology and enzymatic activity. Group I isoforms (NME1-4) have NDPK activity, whereas the members of group II (NME5-9) are more diverse in sequence and possess low or no NDPK activity [12,16]. Human NME1 shares more than 85% sequence homology with NME2 (also known as NDPK-B and NM23-H2) and is reported to function as a hexamer [17,18].

Different crystal structures of apo and substrate-bound (ADP and GDP) forms of NME (NDPK) have been determined, including those from *Homo sapiens*, *Myxococcus xanthus* and *Dictyostelium discoideum* species, among others [19,20]. The NME (NDPK) structure has an α/β domain composed of a central β-sheet (4 anti-parallel β-strands) surrounded by α-helices. The catalytic histidine residue is located within the β-sheet. Two additional structural characteristics are also observed, which involve the Kpn loop and the C-terminal mobile segment [21]. The name of the Kpn loop originated from the “Killer of prune mutation” of the *awd* gene encoding the NDPK of Drosophila [21], and it is reported to contribute to protein surface contacts leading to oligomerization. The C-terminal mobile segment is involved in the dimerization and trimerization of most hexamers. Therefore, both structural characteristics are important for the stabilization of the quaternary structure of the NME protein [22]. The active site, also known as the nucleotide-binding (NB) site, is located within a cleft composed of two helices formed by residues 44–52 and residues 60–70, respectively. Upon substrate binding to the NME2 active site, the F60 of the second helix (residues 60–70) and V112 of the Kpn loop form a clamp, which leads to the stabilization of the base of the substrate.

Human NME1 (hNME1) is reported to be redox-regulated through the oxidation of its cysteine residues both in vitro and in vivo [23,24,25]. During cellular stress, an imbalanced level of reactive oxygen species can lead to the oxidation of protein cysteine residues. In different studies focusing on the oxidation states of hNME1, prolonged exposure to high concentration of hydrogen peroxide leads to irreversible oxidation of C109 to sulfonic acid and an intramolecular disulfide bond formation between C4 and C145 [23,24] (Figure 1A). To prevent overoxidation of proteins, low-molecular-weight thiols (LMW), for example, glutathione (GSH) and coenzyme A (CoA), form a mixed disulfide bond with proteins, which is termed *S*-thiolation (e.g., glutathionylation and CoAlation). Both glutathionylation and CoAlation of hNME1 at C109 inhibit its NDPK activity [24,25]. CoA is a key metabolite that participates in the cellular antioxidant defense mechanism through protein CoAlation [26,27,28,29], which has been reported in stressed bacterial cells (*Staphylococcus aureus*, *Bacillus subtilis* and *Bacillus megaterium*), bacterial spores (*B. subtilis*), stressed HEK293/Pank1β mammalian cells and rat tissues [30,31,32]. Overall, more than 2000 mammalian and bacterial CoAlated proteins have been identified with the use of anti-CoA antibodies together with mass spectrometry (LC-MS/MS) analysis [27,30,33]. The inhibitory effect of CoAlation on the function of different types of proteins has been studied, including CoAlation of *S. aureus* glyceraldehyde 3-phosphate dehydrogenase, human peroxiredoxin 5, *S. aureus* transcription factor AgrA, human NME1, human aurora kinase A and human tau [25,30,34,35,36,37,38,39]. Antioxidant enzymes such as *B. subtilis* thioredoxin A (TrxA) and thioredoxin-like protein YtpP have been shown to deCoAlate proteins through thiol-disulfide reaction mechanisms [40].

The structure of CoA is composed of a 3′-phosphorylated ADP moiety, and a pantetheine tail with a terminal thiol group (Figure 1B) [27]. A detailed study on the regulation of hNME1 by CoA revealed that NME1 is a major CoA-binding protein [25]. Further in vitro studies showed that CoA is capable of binding hNME1 both non-covalently through the nucleotide-binding (NB) site and covalently through binding to the NB site and CoAlating C109 [25]. A structural study of human NME2 in complex with a CoA-derivative, myristoyl-CoA (mCoA) has been reported [41], where mCoA binds non-covalently to the NB site and forms hydrophobic interactions with the adenine ring and salt bridges with the ADP moiety phosphate groups. Following our original findings which revealed NME1 as a major CoA binding protein [25], in this study, we used X-ray crystallography, molecular dynamics and biochemistry to understand the modes of hNME1 regulation by CoA. We solved the high-resolution X-ray crystal structure of hNME1 in complex with CoA and identified residues which contribute to the stabilization of the non-covalently bound CoA within the NB site of hNME1. Using molecular dynamics and an unsupervised learning approach, we characterized the structure of the hNME1-CoA complex, identified key interactions in the complex and verified the high flexibility of the pantetheine tail. Finally, site-directed mutagenesis and CoA-based affinity purifications were used to show that R58 and T94 are important for CoA binding to hNME1. Overall, our data provide a detailed study of the non-covalent mode of CoA binding to hNME1 and reveal the molecular mechanism by which CoA inhibits the hNME1 NDPK activity.

## 2. Results

### 2.1. Purification and Co-Crystallization of hNME1

**Figure 2:**
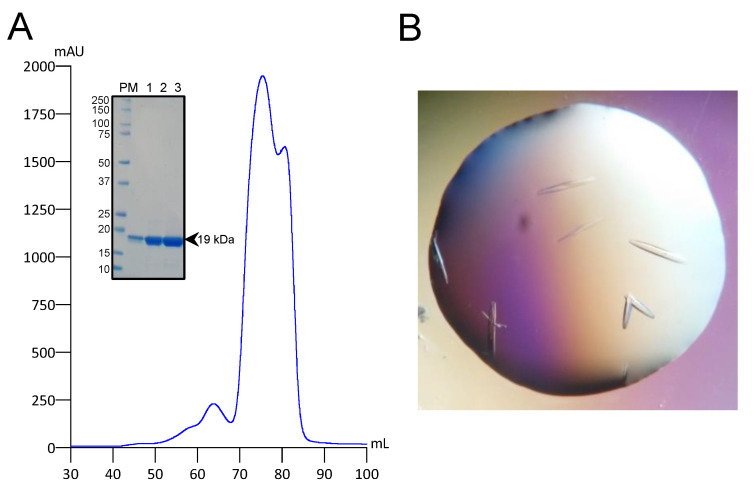
Purification and co-crystallization of hNME1. (**A**) The hNME1 SEC chromatogram is shown, where the blue line represents the absorbance at 280 nm. The purity of the SEC peak fractions was assessed by SDS-PAGE. The insert shows the reducing SDS-PAGE gel with purified hNME1 bands around 19 kDa. The molecular weight (kDa) of the protein markers is indicated. (**B**) hNME1-CoA crystals are shown.

**Figure 3:**
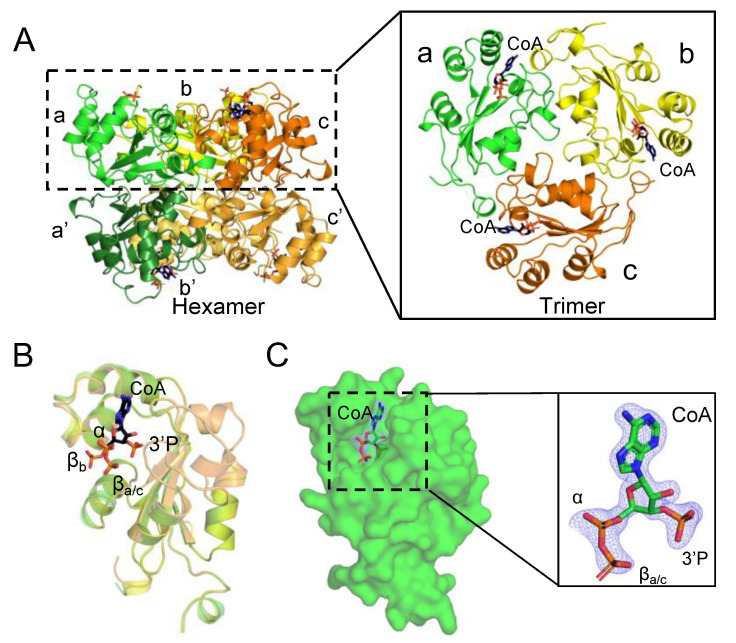
CoA binds non-covalently to the hNME1 nucleotide-binding site. (**A**) The hexameric structure (dimer of trimers) of hNME1-CoA is presented, where the top three monomers (a (green Cartoon), b (yellow Cartoon) and c (orange Cartoon)) forming a trimer are shown in a black dashed rectangle. The bottom three monomers of the hexamer are indicated as a’ (dark green Cartoon), b’ (light orange Cartoon) and c’ (bright orange Cartoon). Each monomer contains a CoA molecule. (**B**) Overlay of the three hNME1-CoA monomers (a, b and c). The α-, β- and 3′-phosphates of CoA are indicated. The β-phosphate of CoA-b has a different orientation than the β-phosphates of CoA-a/c. (**C**) Surface view of hNME1-CoA monomer a. The insert shows the 2Fo-Fc electron density map surrounding the adenosine part of CoA molecule at 1.2σ contour level. The density surrounding the pantetheine tail is not observed. CoA is shown in black or green Sticks and colored by element, where the nitrogen, oxygen, and phosphorous atoms are in blue, red, and orange colors, respectively.

Human NME1 is regulated by the covalent and non-covalent binding of CoA [25]. To expand our understanding of the CoA-mediated regulation of NME1, the wild-type 6xHis-hNME1 was purified using nickel-NTA affinity chromatography (Figure S1) and size-exclusion chromatography (SEC) (Figure 2A). The highly purified protein (12 mg/mL) was co-crystallized with a 10 molar excess of CoA (Figure 2B). Using X-ray crystallography, we solved the CoA bound structure of hNME1 (hNME1-CoA) to a resolution of 1.7 Å and in a space group of I2_1_2_1_2_1_ (Table 1). The overall structure exhibits a topology common to the NDPK domain, consisting of an α/β domain with a central β-sheet with four anti-parallel β-strands, which are surrounded by α-helices. Within the structure, we observed trimers (monomers a, b and c) of the hNME1-CoA complex in the asymmetric unit (Figure 3A). By applying crystallographic symmetries, we obtained the canonical hexameric structure of hNME1-CoA (Figure 3A).

### 2.2. CoA Is Non-Covalently Bound to the Nucleotide-Binding Site of hNME1

CoA contains an ADP moiety (3′-phosphorylated nucleotide, α- and β-phosphates) and a pantetheine tail with a terminal thiol group (Figure 1B). Within our hNME1-CoA structure, CoA is seen unequivocally bound non-covalently to the NB site in each of the three hNME1 monomers (a, b and c) (Figure 3 and Figure S2). Overlay of the monomers a, b and c shows different orientations of the α- and β-phosphates of chain b CoA (CoA-b), compared to CoA of chains a and c (CoA-a/c) (Figure 3B). The 3′-phosphorylated ADP moiety of CoA is observed in all three monomers, but the electron density surrounding the pantetheine tail is absent (Figure 3C and Figure S2). This is likely to be due to the high flexibility of the pantetheine tail, which is solvent exposed.

### 2.3. Stabilization Interactions of CoA within the Nucleotide-Binding Site of hNME1

**Figure 4:**
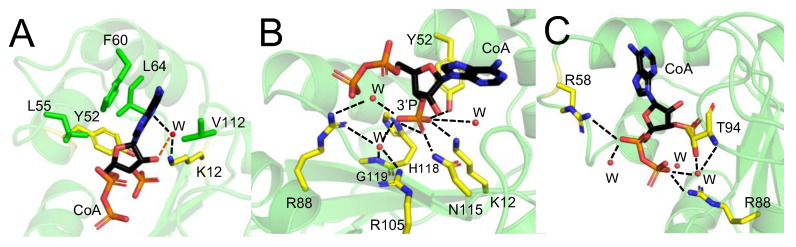
CoA stabilization interactions within the hNME1 nucleotide-binding site. The stabilization interactions of CoA within the NB site of hNME1-CoA monomer a (green Cartoon) are shown (**A**–**C**). The residues that form hydrophobic interactions are colored green, and the residues that form salt bridges or H-bonds (in presence or absence of a water molecule (W)—red sphere) are shown in yellow. (**A**) Interactions between the rings of the ADP moiety of CoA and the hydrophobic patch of the NB site are shown. Interactions between the adenine ring and K12 through a water molecule are shown in black dotted line. H-bond between the hydroxyl group of the ribose ring and a water molecule (W) is shown in orange dotted line. H-bond interactions between the hNME1 NB site residues and (**B**) the 3′-phosphate and (**C**) α-/β-phosphates of CoA are shown in black dotted lines. CoA is shown in Sticks and colored by element, where the carbon, nitrogen, oxygen, and phosphorous atoms are represented in black, blue, red, and orange colors, respectively.

A study analyzing 35 CoAlated protein structures showed that segments of CoA form different types of stabilization interactions with proteins [27]. Hydrophobic interactions are shown to mainly stabilize the rings of the nucleotide moiety of CoA, while phosphate groups mainly form salt bridges with positively charged residues or H-bonds with water molecules. The pantetheine tail is stabilized by alternating polar and non-polar interactions [27]. Within the hNME1-CoA structure, the CoA-a adenine ring is stabilized by F60 via π-π interaction. A hydrophobic patch composed of L55, Y52 (aromatic ring), F60, L64 and V112 stabilizes the adenine and ribose rings of CoA (Figure 4A). The hydroxyl group of the ribose ring interacts with K12 through H-bonding, and the 3′-phosphate group interacts with K12, Y52 (hydroxyl group), N115, H118 and via H_2_O molecules to R88, R105 (side chain) and G119 (main chain) (Figure 4B). CoA α- and β-phosphates are stabilized through interactions with R58 and R88 (salt bridges), T94 (H-bonding through H_2_O molecule with the main chain and side chain) and H_2_O molecules (Figure 4C). Interestingly, the α- and β-phosphates of CoA-b adopt a slightly different conformation than the CoA-a/c, possibly due to the crystal packing. In fact, R58 from a symmetry-related monomer contacts the two phosphates via salt bridges. Important to note is that both Y52 and H118, which are the catalytic residues of hNME1, participate in the stabilization of CoA within the NB site. Unless stated otherwise, the description of the interactions will consider the conformation of CoA-a (hNME1-CoA monomer a) in the rest of the manuscript.

### 2.4. Unique Mode of CoA Binding to the hNME1 Nucleotide-Binding Site

**Figure 5:**
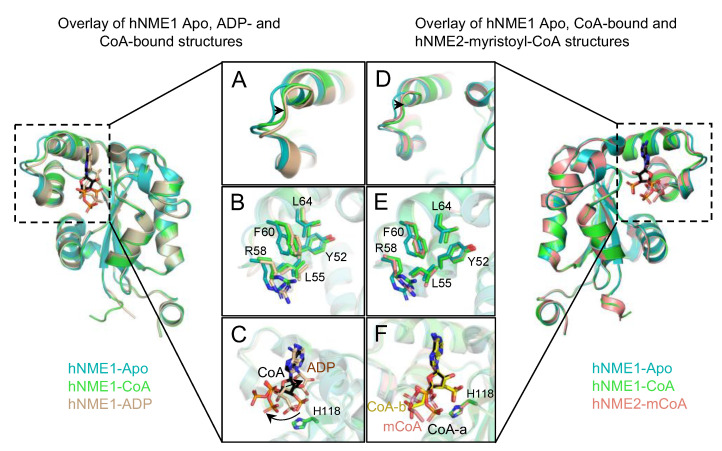
CoA binding induces a local shift of loop56–60 and the hydrophobic patch towards the NB site. Structural overlay of hNME1-apo (cyan Cartoon; PDB: 1JXV), hNME1-CoA (green Cartoon) and hNME1-ADP (wheat Cartoon; PDB: 2HVD) (**A**–**C**) is shown. (**A**,**B**) Residues on the loop56–60 and near the α-helices located directly before and after the loop shift closer to the center of the NB site to stabilize CoA and ADP. The shift is indicated with a black arrow. (**C**) CoA α- and β-phosphates are oriented away from the catalytic center compared to the phosphates of ADP (black arrow). (**D**–**F**) Structural overlay of hNME1-apo (cyan Cartoon; PDB: 1JXV), hNME1-CoA (green Cartoon) and hNME2-mCoA (pink Cartoon; PDB: 7KPF) is shown. (**D**,**E**) Residues on the loop56–60 and near the α-helices located directly before and after the loop shift closer to the center of the NB site to stabilize CoA and mCoA (black arrow). (**F**) mCoA (pink) α- and β-phosphates are also oriented away from the catalytic center, similar to CoA-a (black) and CoA-b (yellow). The CoA-a, CoA-b, ADP and mCoA are colored by element, where carbon atoms are in black, yellow, wheat and pink, respectively. Nitrogen, oxygen, and phosphorous atoms are represented in blue, red, and orange colors, respectively.

We compared the structure of hNME1-CoA with the apo (PDB: 1JXV) and ADP-bound (PDB: 2HVD) forms and observed local structural changes near the NB site. Loop56–60 moves closer to the center of the NB site, as it contains residues R58 and F60, which form a salt bridge with the CoA-phosphates and hydrophobic interaction with the adenine ring, respectively. Residues located on the α-helices directly before (Y52 and L55) and after (L64) the loop also shift towards the center of the NB site (Figure 5A,B). These residues are important for the stabilization of CoA within the NB site (Figure 4). Although the binding of ADP and CoA induce a similar shift in the residues located on loop56–60 and the helices near it, the ribose ring of ADP is located deeper within the NB site and its β-phosphate is oriented towards the catalytic H118 and is buried within the catalytic pocket (Figure 5C). The β-phosphate of CoA is observed to be more solvent exposed and oriented away from the catalytic pocket and H118, while its 3′-phosphate is located near the catalytic H118, where the ATP γ-phosphate would be. Owing to the high resolution of our data, CoA ribose adopts unequivocally a 3′-exo conformation whilst the ADP of hNME1-ADP structure (PDB: 2HVD) adopts a 3′-endo conformation.

A comparison of hNME1-CoA and hNME2-myristoyl-CoA (mCoA) (PDB: 7KPF) structures shows a similar mode of binding within the NB site. Loop56–60 (contains R58 and F60) and the residues within the hydrophobic patch (Y52, L55 and L64) move closer to the center of the NB site to stabilize both CoA and mCoA (Figure 5D,E). The overlay of CoA and mCoA shows that the β-phosphate groups are oriented away from the catalytic H118 (Figure 5F). 

**Figure 6:**
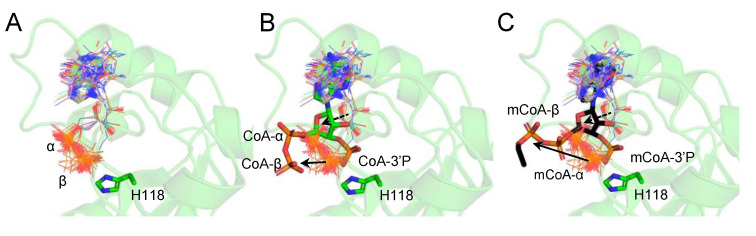
CoA and myristoyl-CoA α- and β-phosphate groups are oriented away from the catalytic pocket. (**A**) Overlay of the nucleotide containing structures of nucleotide kinases. The α- and β-phosphate groups of the nucleotides are oriented towards the catalytic H118. (**B**) CoA and (**C**) myristoyl-CoA (mCoA) α- and β-phosphate groups are oriented away from the catalytic pocket (black arrow). The nucleotide structures are shown in “Lines”, while CoA and mCoA are shown in Sticks. The ribose units of CoA and mCoA are shifted (black dotted arrow) compared to the other bound nucleotides. The nucleotides, CoA and mCoA are colored by element, where carbon atoms are in gray, green and black, respectively. Nitrogen, oxygen, and phosphorous atoms are represented in blue, red, and orange colors, respectively.

We expanded our study to compare the orientation of the α- and β-phosphates of nucleotides within the NB site of nucleotide kinases. Therefore, we retrieved 192 nucleotide kinase structures (EC2.7.4.6) and used only the structures containing nucleotides within their catalytic site (48 structures). We superimposed all the structures to determine the orientation of the α- and β-phosphates (Figure 6A). Interestingly, in all the structures, the phosphate groups of the nucleotides are oriented towards the catalytic H118, while CoA and mCoA bind in a unique way (Figure 6B,C). Their α- and β-phosphate groups are oriented away from the catalytic site towards the solvent. The 3′-phosphate group interacts with the catalytic H118. The ribose rings of the nucleotides are bound deeper within the NB site, compared to the ribose rings of CoA and mCoA (Figure 6B,C), resulting in a shift of about 2.5 Å of the ribose rings.

### 2.5. Molecular Dynamics-Based Characterization of the hNME1-CoA Interaction

**Figure 7:**
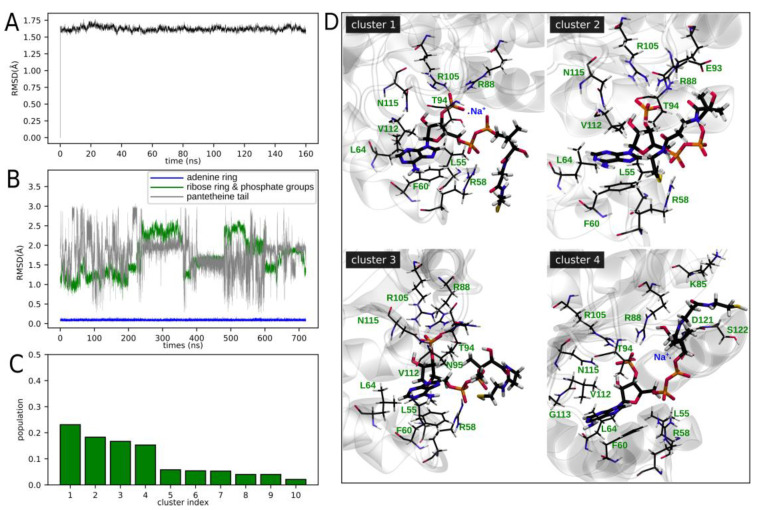
Molecular dynamics-based characterization of the hNME1-CoA interaction in aqueous solution. (**A**) Root Mean Square Deviation (RMSD) of protein atoms along the production run, using as reference the initial structure (which resembles the crystal structure). (**B**) RMSD for different atom groups of CoA, for a stacked trajectory (omitting first 40 ns of the production run, and then stacking the CoA trajectories of each monomer). This way, it should not be interpreted as a time trace formally, but as an indicator of the different atom groups structural fluctuations. (**C**) Populations of the 10 clusters found with the k-means clustering method, performed on the stacked CoA trajectory. (**D**) Representative structures of the four most populated clusters, and surrounding protein residues (green labels) and sodium ions (blue labels) closer than 3 Å from any CoA atom. Hydrogen, carbon, nitrogen, oxygen, and phosphorus atoms are represented in white, black, blue, and red colors, respectively.

Using molecular dynamics (MD) simulations, we aimed to characterize the hNME1-CoA complex in an aqueous solution, focusing our attention on the local environment of CoA (Figure 7). The MD studies were performed using the crystal structure reported above (PDB: 8OOV—Figure 3) as a starting point. After the equilibration of the system, a production MD run was performed at room temperature (RT), and the complex dynamic structure was assessed. Figure 7A shows the evolution of the Root Mean Square Deviation (RMSD) along the production run, for the protein atoms. The reference structure corresponds to the first structure (before equilibration) and resembles the crystal structure. It can be seen that the RMSD value quickly reaches a *plateau* and remains lower than 2 Å, showing a rigid protein structure, as previously reported for the nucleotide diphosphate kinase (NDK) protein of *Porphyromonas gingivalis*, which also exhibits nucleotide-binding capability [42]. On the other hand, when analyzing the CoA RMSD (Figure 7B) on the stacked MD trajectory, despite the adenine ring staying still, with almost constant RMSD values, atoms belonging to the ribose and phosphate groups show significant fluctuation. This is even more pronounced for the pantetheine tail. Regardless of being a stacked trajectory which combined the sampling obtained from the six monomers (and for this reason should not be interpreted as a temporal trace), still retrieves valuable information and points out the high flexibility exhibited by the CoA molecule (or part of it), even when bound to hNME1. These results are in agreement with the lack of electron density associated with the pantetheine tail in the hNME1-CoA X-ray crystal structure.

While part of the hNME1-CoA interactions can be characterized from the crystal structure, as we showed in the previous sections, the key interactions between hNME1 and the pantetheine tail of CoA cannot be assessed in the same way due to the absence of electronic density (Figure 3 and Figure S2). To achieve this goal in an unbiased manner, we employed an unsupervised learning approach, performing a k-means clustering on the CoA stacked trajectory (searching a maximum of 10 clusters—Figure 7C,D). In Figure 7C, the cluster’s population exhibits a drop after the fourth cluster. Even more, about 75% of the structures fall in clusters 1 to 4, while the rest of them are spread in other clusters. In the following, we will focus our attention on the four most populated clusters. In Figure 7D, the interactions between the CoA molecule and its surroundings are studied, using representative structures of each of the selected clusters. Interactions with certain hNME1 residues are conserved in all clusters. In agreement with the crystal structure, this includes the interaction between F60 and the adenine ring and the residues L55, L64 and V112, which all together constitute the hydrophobic pocket of the NB site. Additionally, the interaction between 3′-phosphates and R88, T94 and R105, as well as R58 with α-phosphate, are also conserved in every cluster.

The most distinctive pattern in each cluster is given by the interactions involving the β-phosphate and the pantetheine tail. Interestingly, Na^+^ ions used in the simulation to neutralize the system can be seen stabilizing CoA bound to NME1. Clusters one and four show that Na^+^ is coordinated by the β- and 3′-phosphates of CoA. This, in turn, imposes a restriction on the diphosphate group orientation and consequently on the pantetheine tail orientation. For instance, when comparing cluster one with cluster three, in the former the pantetheine tail remains quite exposed to the solvent, but in cluster three, it is able to bend, establishing hydrophobic-like interactions with V112 and T94, through the methyl groups. In cluster two, the pantetheine tail is stabilized with intramolecular interactions between its amino groups, the thiol group and the β-phosphate and also exhibits interactions with surrounding residues. Particularly, interactions with E93 (carboxylate group with CoA hydroxide group) and T94 (amide group with CoA carbonyl group) are observed. In the case of cluster four, an interesting packaging of the pantetheine tail is observed. The coordination of 3′-phosphate to Na^+^ induces a shift in R88, which seems to induce a pocket-like arrangement of residues L85, D121 and S122, in which the pantetheine tail lays.

Remarkably, a control simulation employing a different forcefield (CHARMM27 [43] instead of amber ff14SB [44]) retrieved an excellent agreement in terms of interactions stabilizing the adenine and ribose rings and the phosphate groups, where the complex stabilization comes from non-covalent interactions with residues R55, R58, F60, L64, R88, T94, R105, V112 and N115. Additionally, interactions between the pantetheine tail -OH group and residues K12 and D54 were observed. From a more qualitative perspective, both forcefields agreed on the flexible properties of the pantetheine tail, and that coordination to Na^+^ favors different accommodations of the CoA molecule within the protein environment.

### 2.6. Mutation of R58 and T94 Disrupts hNME1 Binding to CoA

**Figure 8:**
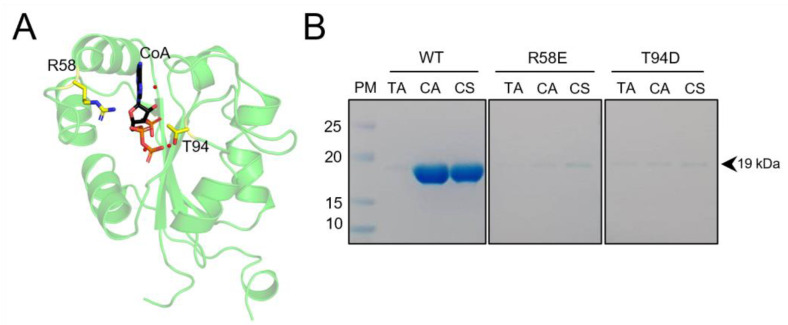
R58 and T94 are important for CoA binding to the hNME1 nucleotide-binding site. (**A**) The hNME1-CoA structure is presented in green Cartoon, and the R58 and T94 residues in yellow Sticks. CoA is colored by element, where the carbon, nitrogen, oxygen, and phosphorous atoms are represented in black, blue, red, and orange colors, respectively. (**B**) The SDS-PAGE gel analysis of affinity purifications of hNME1 WT, R58E and T94D mutants is shown. Three types of matrices were used to assess the hNME1 binding, Tris-agarose control beads (TA), CoA-agarose (CA) and CoA-sulfolink (CS). WT hNME1 binds strongly to both CoA-agarose and CoA-sulfolink but does not bind to the Tris-agarose control matrix. Mutation of R58 and T94 disrupts the binding of hNME1 to CoA. The molecular weight of hNME1 WT and mutants (around 19 kDa) is indicated. At least three independent replicates were performed.

From our data on the hNME1-CoA stabilization study, we observed that R58 and T94 are important for the stabilization of CoA within the hNME1 catalytic pocket (Figure 8A). Furthermore, a recent study from our laboratory revealed selective binding of CoA to aurora A kinase, involving the interaction between the 3′-phosphate ADP moiety of CoA and T217 located in the ATP binding site [39]. A combination of biochemical, biophysical, and crystallographic approaches showed that the 3′-phosphate group of CoA forms a H-bond with the side chain of the evolutionarily conserved T217 in aurora A kinase, while the interaction is disrupted with the T217E mutant. Therefore, we generated hNME1 R58E and T94D mutants by Phusion site-directed mutagenesis and purified the proteins. The binding of the hNME1 WT and mutants was assessed. Two types of CoA-based affinity matrices were used, where CoA was either immobilized through the thiol group (CoA-sulfolink) or the amino group of the adenine ring (CoA-agarose). The Tris-agarose matrix was used as control. The proteins (0.15 mg) were incubated with the different matrices for 1 h at 4°C. The matrices were then washed and boiled in the presence of an SDS-loading buffer, and SDS-PAGE was used to assess protein binding to beads. The WT hNME1 showed strong binding to both CoA-sulfolink and CoA-agarose matrices, while background non-specific binding was observed to the Tris-agarose control matrix (Figure 8B). On the other hand, the binding of R58E and T94D mutants to the CoA-matrices was disrupted (Figure 8B). This highlights the importance of both residues mediating specific interaction of CoA within the NB site of hNME1.

## 3. Discussion

CoA is a LMW thiol which participates in diverse cellular functions including the antioxidant defense system [28,29]. Recent studies show that over 2000 proteins from different model organisms are covalently modified by CoA under oxidative stress conditions [27,30,31]. The majority of these proteins are involved in cellular metabolic processes (around 60%), stress response and protein synthesis [27], showing the important cellular regulatory role CoA plays in cellular response to oxidative stress [28,29,45]. In our previous study [25], we focused on identifying proteins that non-covalently bind to CoA using CoA-based affinity matrices (CoA-sulfolink and CoA-agarose). Affinity purification together with mass spectrometry analysis showed that hNME1 is a major CoA-binding protein. In the same study, we showed that hNME1 is regulated by CoA through covalent and non-covalent binding. Biochemical studies showed that CoA is a competitive inhibitor of the nucleoside kinase activity of hNME1 [25]. Here, X-ray crystallography, biochemistry and molecular dynamics were used to investigate the mode of CoA binding to the hNME1.

hNME1 is regulated by diverse oxidative post-translational modifications, including sulfonylation, glutathionylation and CoAlation of C109 and disulfide bond formation between C4 and C145 [23,24,25]. These modifications were shown to inhibit the nucleoside diphosphate kinase activity of hNME1. Comparison of the crystal structures of the apo and oxidized forms of hNME1 reveal that upon oxidation, a disulfide bond is formed between the C4 and C145, which solvent exposes C109 located on the Kpn loop (Figure 1A) within the catalytic site [24]. In a separate study, C109 was reported to be CoAlated in the presence of oxidizing agents, suggesting that following disulfide bond formation (C4-C145) and exposure of C109, CoAlation of redox-sensitive C109 could occur [25]. Here, we used X-ray crystallography and molecular dynamics to investigate the non-covalent binding of CoA to hNME1. The structure of hNME1-CoA complex revealed CoA bound to the NB pocket of hNME1, where the α- and β-phosphates and the “pantetheine tail” (not visible within the structure) are oriented away from both the catalytic pocket and the C109. This shows that there is a distinct mode of CoA binding to hNME1 under different cellular conditions.

CoA thioesters are intermediates of important biochemical pathways which are central for cellular metabolism. Recently, the long-chain fatty acyl (LCFA) CoA-mediated regulation of hNME2, which has a very similar protein sequence and structure to hNME1, was studied [41]. Biochemical studies showed that the binding of LCFA-CoA inhibits the nucleoside diphosphate kinase activity of hNME1 and hNME2. By solving the structure of hNME2 bound to a LCFA-CoA (e.g., myristoyl-CoA), residues important for the stabilization of myristoyl-CoA to hNME2 were identified. A comparative study of the myristoyl-CoA and GDP-bound structures of hNME2 showed that R58 is important for the stabilization of myristoyl-CoA phosphate groups but does not interact with GDP. With further biochemical assays, the R58 mutation was shown to maintain the hNME2 NDPK activity but weakened the interaction with LCFA-CoA [41]. Here, we showed that hNME1 R58 forms a salt bridge with CoA phosphates, and its mutation prevents binding to CoA (Figure 4 and Figure 8B). The comparative study between CoA and ADP-bound hNME1 also showed that R58 stabilizes the phosphates of CoA, but not ADP (Figure 5). In a different study, the hNME2 T94D and L64E mutants were shown to be catalytically inactive [41]. The mouse NME1 T94D was shown to be catalytically less active than the WT [46]. Both T94 and L64 within the hNME1-CoA structure were shown to stabilize CoA (Figure 4), and mutation of these residues to negatively charged residues could interfere with substrate binding, as was observed in our CoA-based affinity purifications with T94D mutated protein (Figure 8).

A study performed on CoAlated protein structures suggests different modes of CoA binding to proteins [27]. These include the initiation of CoAlation through either the stabilization of the ADP moiety (adenine ring or the phosphate groups) or the pantetheine tail. This initial interaction would prime the other segments of CoA to interact with the protein, leading to a disulfide bond formation. Within this study, we have shown that both R58 and T94, which stabilize the phosphate groups of CoA are crucial for the hNME1 binding to CoA. The same study also focuses on different stabilization interactions CoA forms with proteins, which is coherent with the interactions CoA forms with hNME1. A hydrophobic patch stabilizes the adenine and ribose rings of CoA, the phosphates are stabilized by salt bridges or H-bonds via water molecules [27]. By solving the structure of hNME1 in complex with CoA, we determined key residues that are important for the CoA-mediated regulation of hNME1. In line with these observations, the molecular dynamics simulations demonstrate that the core of CoA remains firmly bound to hNME1 with interactions involving positively charged (R58, R88, T94 and R105) and hydrophobic (L55, L64, F60 and V112) residues. The molecular dynamics experiments demonstrate the high flexibility of the CoA pantetheine tail, explaining the absence of electronic density in the X-ray crystal structure. Nevertheless, we were able to observe more favorable conformations that the CoA pantetheine tail can adopt. The tail can be stabilized by residues K12, D54, L85, T94, V112, D121 and S122. Interestingly, the presence of Na^+^ seems to influence the conformational landscape explored by the pantetheine tail. Overall, our results reveal a unique mode by which CoA binds to the NB site of hNME1, which differs significantly from that of ADP binding.

## 4. Materials and Methods

### 4.1. Construction of hNME1 R58E and T94D Mutants

hNME1-pET28a(+) plasmid [25] was used to generate the R58E and T94D mutations by using Phusion site-directed mutagenesis with phosphorylated (p) primers. Forward primer 5′-p-GAACCCTTCTTCGCGGGTTTAGTAAAG-3′ and reverse primer 5′-p-ATCTTTAAGATCCAC ATAATGCTCTTTCAGT-3′ were used to generate R58E mutation, forward primer 5′-p-GATAATCCGGCGGATAGTAAACCG-3′ and reverse primer 5′-p-TTCTCCCAACATAACACGACCAG-3′ were used to generate T94D mutation. Following PCR amplification, the linear PCR product was circularized using the T4 DNA ligase (New England Biolabs Ltd, Hitchin, UK). The plasmid was then transformed into *E. coli* Top10 cells. Sequencing was used to confirm the R58E and T94D mutations.

### 4.2. Expression and Purification of hNME1 Wild-Type, R58E and T94D Mutants

BLR(DE3) cells containing 6xHis-hNME1-pET28a(+) WT, R58E and T94D plasmids were grown at 37 °C in Luria Broth (Sigma-Aldrich, Gillingham, UK) media until OD_600_ reached 0.8. The cells were induced with Isopropylthio-β-galactoside (IPTG—0.5 mM) and incubated overnight (O/N) at 25 °C. Centrifugation (15 min at 6200× *g* at 4 °C—Beckman JLA 8.1000 rotor) was used to harvest cells and the pellet was resuspended in 50 mM Tris pH 7.5, 0.5 M NaCl, 5 mM imidazole (Millipore, Watford, UK), 1 mM beta-mercaptoethanol (Sigma-Aldrich, Gillingham, UK), 50 μg/mL deoxyribonuclease I (DNaseI Bovine pancreas—Sigma), 1x PIC (cOmplete^TM^ Mini Protease Inhibitor Cocktail—Roche) and 10 mM MgCl_2_. Sonication was performed using Soniprep150 (15 cycles—15 s pulse ON/20 s pulse OFF), followed by centrifugation (40 min at 39,000× *g* at 4 °C using Beckman JA25.50 rotor). The cell lysate was then filtered, and in-batch incubated with super nickel-NTA affinity beads (Neo Biotech, Nanterre, France) equilibrated in binding buffer (50 mM Tris pH 7.5, 0.5 M NaCl, 1 mM beta-mercaptoethanol and 10 mM MgCl_2_) for 1 h at 4 °C (with gentle rotation). The beads were then packed in an empty Xk16/20 column (Cytiva, Amersham, UK), and the column was connected to the ÄKTA Start purification system (Cytiva, Amersham, UK). A linear gradient elution (0–100% elution buffer) to 0.5 M imidazole was used to elute the bound protein. The fractions which showed increase in absorbance at 280 nm were collected and their purity was assessed using SDS-PAGE.

Size exclusion chromatography (SEC) was used to further purify the proteins. The protein sample obtained after affinity purification was concentrated and injected into a HiLoad™ 16/600 Superdex™ 200 pg (Cytiva) column equilibrated with 50 mM Tris pH 7.5, 0.5 M NaCl, 10 mM MgCl_2_ and 5 mM DTT. The fractions which showed increase in absorbance at 280 nm were collected and their purity was assessed using SDS-PAGE. The pure samples were stored with 5% glycerol and 5 mM DTT at −20 °C.

### 4.3. hNME1-CoA Complex Co-Crystallization, X-ray Data Collection and Structure Determination

Prior to crystallization, hNME1 was repurified using a Superdex75 10/300 increase equilibrated in 20 mM HEPES pH 7.0 and 100 mM NaCl buffer and concentrated to 18 mg/mL using 10 kDa cut-off Vivaspin concentrators. hNME1 (12 mg/mL) was incubated at room temperature (RT) with 10 molar excess of CoA. Crystals were obtained by mixing 1 µL of the complex with 1 µL of 26–30% MPD (2-methyl-2,4-pentanediol) and 100 mM MES (2-(*N*-morpholino)-ethane sulfonic acid) pH 6.5 at room temperature. The obtained crystals were directly frozen in liquid nitrogen, and the diffraction data were collected on ID30A-1. The data were processed using XDS [47] and aimless [48]. The structure was solved by molecular replacement using Phaser-2.7.0 [49] and hNME1-ADP complex structure (PDB: 2HVD) without ADP was used as a search model. The refinement was carried out with Buster 2.10.4 (Global Phasing, Cambridge, UK) [50] and Coot 0.9.3 [51]. The composite omit map was calculated with Phenix 1.20.1-4887 [52]. The data collection statistics and refinement parameters are summarized in Table 1.

### 4.4. Affinity Purification of hNME1 WT, R58E and T94D Mutants Using Coenzyme A-Based Affinity Matrices

Two types of CoA-based affinity matrices, CoA-sulfolink and CoA-agarose, were used. CoA-sulfolink was prepared by coupling CoA (Sigma-Aldrich, Gillingham, UK) to the SulfoLink^®^ Coupling Resin using the manufacturer’s instructions (Thermo Scientific™, Rockford, IL, USA). The lyophilized CoA-agarose (Sigma-Aldrich) and Tris-agarose (control) matrices were generated by adding 5× bead volume of expansion buffer 1 (50 mM Tris pH 8.0 and 30 mM NaCl) and buffer 2 (500 mM Tris pH 8.0), respectively. All matrices were then washed 3× with 50 mM Tris pH 8.0 and 30 mM NaCl buffer.

Prior to the start of the assay, the purified hNME1 WT, R58E and T94D proteins were reduced with 10 mM DTT and desalted using HiTrap desalting column (Cytiva) equilibrated in 50 mM Tris pH 8.0 and 30 mM NaCl. The column was connected to the ÄKTA Start purification system (Cytiva). The three matrices were incubated with 150 µg of hNME1 WT, R58E and T94D mutants for 1 h at 4 °C (with rotation). The matrices were then washed 3× with 50 mM Tris pH 8.0 and 30 mM NaCl and centrifuged (1000× *g*, 2.5 min at 4 °C). After the final centrifugation, the supernatant was removed, and the beads were resuspended in SDS-PAGE protein loading dye (LD). The samples were boiled, centrifuged and the supernatant was loaded on SDS-PAGE gel. The gel was stained using InstantBlue^TM^ Coomassie staining. Three independent replicates of this experiment were performed.

### 4.5. Molecular Dynamics of the CoAlated hNME1

A classical molecular dynamics (MD) approach was used to investigate key ligand–protein interactions in the hNME1-CoA complex. The structural starting point was the PDB file obtained from the X-ray crystal structure of the hNME1-CoA reported in this work, which corresponds to the hNME1 hexamer non-covalently bound to CoA. The parameters for the CoA molecules were obtained with antechamber and parmcheck [53], with restrained electrostatic potential (RESP) charges fitted from an in vacuo Hartree-Fock/6-31G* [54] single point calculation (using Gaussian09) [55], on a manually generated structure for CoA (generated with MOLDEN) [56]. The chosen forcefields were ff14SB and the Generalized Amber Forcefield (gaff), for the protein and CoA molecules, respectively. Any molecule different from CoA or the protein itself was removed using the prepareforleap package. Missing atoms were added using LEAP [53] (including hydrogen atoms and the unsolved pantetheine tail). The protonation states of ionizable residues were determined using propKa [57] (pH = 7.4). All histidine residues were protonated on the epsilon nitrogen, since no specific interactions suggesting alternative protonation states were found. Sodium ions were added to neutralize the originally negatively charged system. The hNME1-CoA complex was solvated with 17,502 water molecules, in a TIP3P [58] truncated octahedral box, using LEAP. 

The MD simulations were carried out with AMBER16 [53] employing periodic boundary conditions with a 10 Å cutoff and particle mesh Ewald summation method for treating the electrostatic interactions. The SHAKE algorithm was used to keep hydrogen bond lengths at their equilibrium distance. The simulation was started by applying quadratic restraints (first structure as reference) to selected atom groups. After a short minimization to eliminate possible clashes, the system was heated to 300 K in a stepwise manner, releasing the restraints and increasing the time step gradually, in the NVT ensemble (e.g., constant volume, temperature and amount of substance) with the Berendsen thermostat [59]. Then, the system density was adjusted in 10 ns in the NPT ensemble (analogous to NVT, but with constant pressure instead of volume). Finally, 160 ns long unrestrained MD was performed, using a time step of 2 fs, and the Langevin thermostat [60] to keep the temperature around 300 K. Since the protein root mean square deviation (RMSD, calculated with CPPTRAJ) [61] reached a steady value in about 40 ns, only the final 120 ns of the simulation were used for analysis. 

The ligand–protein interaction was assumed to be dynamically equivalent for the six monomers, and so the MD trajectories of CoA molecules were stacked and analyzed together (resulting in a 720 ns long trajectory). After aligning the stacked CoA trajectory to the first frame, RMSD calculations were performed for different atom groups from CoA. Additionally, to better characterize the ligand–protein interaction, a cluster analysis based on the CPPTRAJ implementation of k-means clustering algorithm [62] was performed. Only the four most populated clusters were considered for analysis (which together represented about 75% of the total data). Representative structures were obtained for each of them, searching in each case the structure closest to the cluster centroid. Finally, a control MD simulation using the CHARMM27 forcefield was performed starting with a random frame from the ff14SB trajectory. The system was built starting with the coordinates of non-solvent molecules, reconstructing the solvent box, using gmx, from the GROMACS package [63]. Then, the topology file was converted to amber-like parameters using PARMED [64]. The simulations were carried out with AMBER with an identical equilibration protocol to that of the ff14SB case, followed by a 20 ns long NVT production run to reach steady RMSD values for the protein residues. Then, a 100 ns long stacked CoA trajectory was generated, and the k-means clustering analysis was performed this time aiming directly for the four most populated clusters. Key interactions were determined again based on the structures closer to each cluster centroid. All the visualizations were performed with VMD.

## Figures and Tables

**Table 1:** X-ray data collection and refinement statistics for the hNME1-CoA structure.

Dataset	hNME1-CoA
**PDB ID**	**8OOV**
**Data collection**	
Beamline	ID30A-1, ESRF
Wavelength (Å)	0.965459
**Processing**	
Space group	I2_1_2_1_2_1_
Cell parameters (Å/°)	79.1 113.0 118.1 90 90 90
Resolution (Å)	43.6–1.70 (1.73–1.70) *
Total reflections	289,457 (14,472) *
Unique reflections	58,147 (3052) *
Unique reflections	58,147 (3052) *
Rmerge	0.087 (0.971) *
Rpim	0.066 (0.750) *
Redundancy	5.0 (4.7) *
I/s(I)	9.2 (1.4) *
Completeness (%)	99.6 (99.8) *
CC_1/2_	0.996 (0.505)*
**Refinement**	
Resolution range (Å)	23.66–1.7
Rwork/Rfree (%)	16.95/19.59
No. atoms	
Protein	3694
Ligand (CoA)	93
Water	514
B-factors	
Protein	24.8
Ligand (CoA)	33.6
Water	41.4
R.m.s deviation	
Bond lengths (Å)	0.009
Bond angles (°)	0.97
Ramachandran Plot	
Favored/allowed/outliers (%)	98.48/0.87/0.65

* Highest resolution shell is shown in parenthesis.

## Data Availability

Data are contained within the article or Supplementary Material.

## Supplementary Materials

The following supporting information can be downloaded at: https://www.mdpi.com/article/10.3390/ijms24119359/s1.
