# Supplementary material for: A Unique Mode of Coenzyme A Binding to the Nucleotide Binding Pocket of Human Metastasis Suppressor NME1
Source: Int J Mol Sci. 2023 May 27;24(11):9359. doi: 10.3390/ijms24119359 (PMC10253429; doi:10.3390/ijms24119359)
Supplement: Supplementary file 1 [file ijms-24-09359-s001.zip › ijms-2364669-supplementary.pdf]

Supplementary Figures

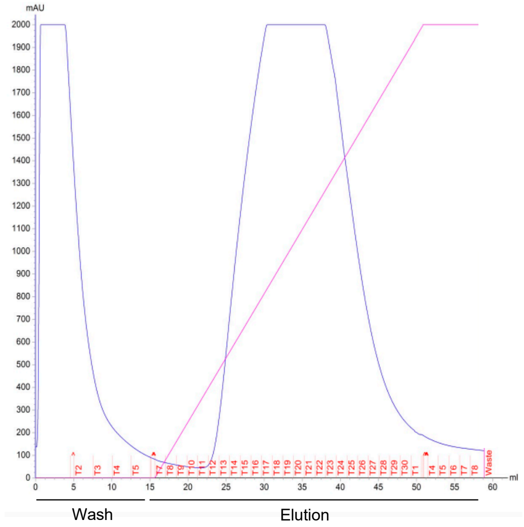

Figure S1. Affinity purification of hNME1. The hNME1 nickel-NTA affinity purification chromatogram is shown. The blue line represents the absorbance at 280 nm, and the pink line represents the linear gradient elution of hNME1 from 0 to 100% imidazole.

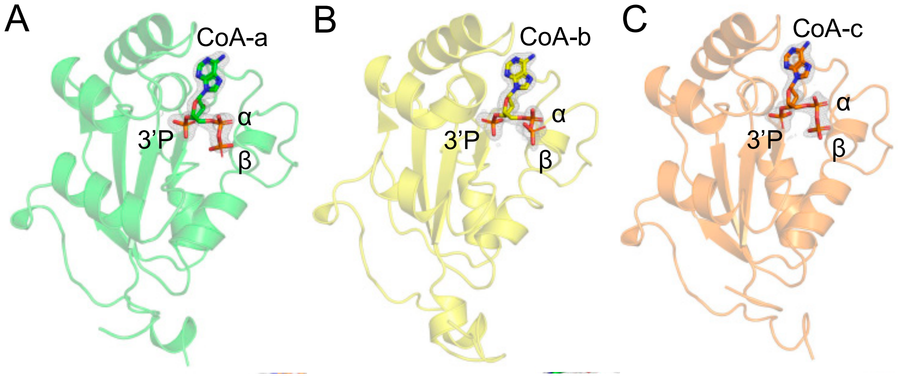

Figure S2. Composite omit maps of the hNME1-CoA monomers. The structure of hNME1-CoA monomers (**A**) a, (**B**) b and (**C**) c are shown. The gray Fo-Fc omit maps surrounding CoA at contoured level 1.5σ are shown. Electron density (gray) is observed surrounding the 3′-phosphorylated ADP moiety but is absent for the pantetheine tail. CoA is shown in green (**A**), yellow (**B**) or orange (**C**) Sticks and is colored by element, where the nitrogen, oxygen, and phosphorous atoms are in blue, red and orange, respectively.
